# Genome-Wide Identification and Expression Profiling of Phosphatidylethanolamine-Binding Protein (PEBP) Genes in *Helianthus annuus* L.

**DOI:** 10.3390/ijms26104602

**Published:** 2025-05-11

**Authors:** Yiyi Sun, Yanwen Wang, Jingyan Bai, Jiatong Guo, Guiting Li, Qiuzhen Tian, Shuping Lv, Hengchun Cao, Xiaojie Yang, Lingyun Liu

**Affiliations:** 1 School of Life Sciences, Henan University, Kaifeng 475001, China; suimuyinyang01@163.com (Y.S.); wendtydty111@163.com (Y.W.); b2455515414@163.com (J.B.); 2221010075@henu.edu.cn (J.G.); 2 Henan Key Laboratory of Specific Crops Genomics, Henan Sesame Research Center, Henan Academy of Agricultural Sciences, Zhengzhou 450002, China; liguiting07111106@163.com (G.L.); tianqiuzhen@163.com (Q.T.); hncotton6356@163.com (S.L.); chczhima@163.com (H.C.); 3 Henan Joint Key Laboratory of Specific Oilseed Crops, Zhengzhou 450002, China; 4 Henan Sesame Research Center, Henan Academy of Agricultural Sciences, Zhengzhou 450002, China; 5 Economic Crop Research Institute, Henan Academy of Agricultural Sciences, Zhengzhou 450002, China

**Keywords:** expression profiles, gene duplication and evolution, PEBP gene family, phylogenetic analysis, sunflower

## Abstract

The phosphatidylethanolamine-binding protein (PEBP) gene family is critical for regulating plant growth, development, and flowering. Sunflower (*Helianthus annuus* L.) is the fourth most important oilseed crop globally. However, the genomic structure and functional diversity of *PEBP* genes in sunflower remain unexplored. Leveraging the recently assembled telomere-to-telomere (T2T) sunflower genome, a genome-wide analysis of the *HaPEBP* family was carried out. A total of 12 *PEBP* genes were identified in sunflower and categorized into three subfamilies: TFL1-like, FT-like, and MFT-like. Phylogenetic and synteny analyses revealed that tandem duplication events have substantially contributed to the evolution and expansion of the *HaPEBP* gene family. Furthermore, the analysis of the promoter regions revealed 77 distinct *cis*-acting elements, including 35 related to light signaling and growth regulation, highlighting their potential involvement in the regulation of flowering and development in sunflower. Expression profile analysis using RNA-seq data across various tissues indicated that FT-like and TFL1-like *HaPEBP* genes may be the key regulators of flowering time and plant architecture in sunflower varieties. This study offers valuable insights into the structural, evolutional, and functional dynamics of the *HaPEBP* gene family and holds significant implications for sunflower breeding strategies aimed at optimizing flowering time and plant architecture traits.

## 1. Introduction

The phosphatidylethanolamine-binding protein (*PEBP*) gene family represents an ancient and highly conserved group of genes present across all eukaryotic kingdoms, including bacteria, animals, and plants [1,2]. In plants, *PEBP* genes serve as key regulators of diverse developmental processes, including flowering time, plant architecture, and responses to environmental stress [3]. These genes encode proteins that participate in multiple signaling pathways essential for plant growth and differentiation. Although the functions of specific *PEBP* genes have been widely studied, comprehensive analyses of the entire *PEBP* gene family have been carried out in only a limited number of species, such as *Arabidopsis*, soybean, rice, tomato, barley, and pepper [4,5,6,7,8].

The *PEBP* gene family comprises three main subfamilies: *FLOWERING LOCUS T* (*FT*)-like, *TERMINAL FLOWER1* (*TFL1*)-like, and *MOTHER OF FT AND TFL1* (*MFT*)-like, each with distinct roles in plant growth and reproductive transitions [3]. FT-like proteins, including *FT* and its close homolog *TWIN SISTER OF FT* (*TSF*), serve as primary activators of flowering [9]. FT, often referred to as the florigen, acts as a mobile signal that travels from the leaves to the shoot apical meristem (SAM), where it interacts with the transcription factor *FLOWERING LOCUS D* (*FD*) to induce flowering [1,10]. *TSF* carries out a similar function; its overexpression accelerates flowering, whereas TSF-deficient mutants exhibit delayed flowering [11]. Conversely, TFL1-like proteins, including *TFL1*, *BROTHER OF FT AND TFL1* (*BFT*), and *CENTRORADIALIS* (*CEN*), function as floral repressors [12]. *TFL1* is primarily expressed in the inflorescence meristems, where it negatively regulates inflorescence development. Mutations in *TFL1* result in accelerated flowering, emphasizing its role as a flowering inhibitor [1]. *BFT*, which exhibits diurnal expression patterns, delays flowering when overexpressed, although its knockdown has no significant effect, indicating functional redundancy within this subfamily [13]. In contrast, the *MFT*-like subfamily demonstrates attenuated *FT*-like activity and is primarily involved in seed germination and responses to environmental stress [14,15]. Interestingly, despite their high DNA sequence similarity, *FT* and *TFL1* have opposing effects on flowering, reflecting the intricate regulatory mechanisms and evolutionary divergence within the *PEBP* family [16]. It is hypothesized that *MFT*-like genes may be ancestral to both *FT*-like and *TFL1*-like subfamilies, contributing to their functional diversification in plant developmental processes.

Elucidating the functions of the *PEBP* gene family is crucial for crop improvement, especially in relation to flowering time and plant architecture, two key factors that influence yield [17,18]. For instance, in rice, the florigen gene *Hd3a* exhibits approximately 70% amino acid sequence similarity with *FT* and 50% with *TFL1* from *Arabidopsis*, and it enhances branching while promoting flowering [19]. In sesame, the *TFL1*-like gene *SiPT1* significantly influences plant architecture; its overexpression results in increased branching while delaying flowering [20]. Similarly, in pepper (*Capsicum* spp.), two *FT*-like genes, *CaFT5* and *CaFT6*, have been identified as key regulators of flowering time and plant structure [8]. Collectively, these findings highlight the diverse and vital roles of the *PEBP* gene family in regulating both plant growth and reproductive processes.

Sunflower (*Helianthus annuus* L.) is one of the most recognizable members of the Asteraceae family, which accounts for approximately 10% of all flowering plant species. It is the fourth most important oilseed crop globally, following palm, soybean, and rapeseed [21,22]. Furthermore, sunflower holds economic importance in the cut flower industry. Enhancing seed yield from terminal flower discs and improving terminal flower quality are the primary goals in both oilseed and ornamental sunflower breeding programs [23]. Plant architecture, specifically the distinction between uniculm and branched types, is a critical trait in sunflower cultivation. The presence of side buds and branches can negatively affect terminal flower development and increase production costs [23]. Consequently, developing new varieties with reduced or no lateral branching is vital for increasing seed yield. In ornamental sunflowers, branching patterns also play a critical role in determining flower quality and yield. Previous research has explored the role of recently duplicated *FT* paralogs in the sunflower genome during domestication, particularly focusing on the *FT*/*TFL1* gene family [24,25]. These studies have revealed that modifications in gene expression, sequence variation, and gene interactions, notably a frameshift mutation in *HaFT1*, which is associated with a major QTL for flowering time, can delay flowering by interfering with *HaFT4* [24,25]. These findings emphasize the importance of *FT* paralogs in driving adaptive changes, varied photoperiod responses, and evolutionary innovation through gene duplication. Despite these insights, the genome-wide characteristics and roles of *PEBPs* have not been systematically investigated in sunflower.

Thus, in this study, we conducted a comprehensive genome-wide identification and expression analysis of the *HaPEBP* gene family, employing bioinformatics approaches based on a self-constructed sunflower telomere-to-telomere (T2T) genome. The investigation encompassed an in-depth analysis of gene structure, selection pressure, and comparative evolutionary patterns within the *HaPEBP* family. The study also examined the physicochemical properties of *HaPEBP* proteins and predicted their 3D structural models. Furthermore, protein–protein interactions and potential regulatory networks were assessed using the STRING database. To further elucidate the functional roles of *HaPEBP* genes, their transcriptional profiles across various tissues were analyzed using RNA-seq data. These comprehensive analyses lay a robust foundation for understanding the biological functions and molecular regulatory mechanisms of the *HaPEBP* family, offering valuable insights into their functions in sunflower growth and development.

## 2. Results

### 2.1. Genome-Wide Identification and Phylogenetic Relationship of HaPEBP Members in Sunflower

Based on BLAST(version 2.8.1) and HMM (v3.3.2) searches, a total of 12 *PEBP* genes were identified in the T2T-assembled genome of sunflower, each containing the complete PEBP domain (Supplementary Table S1). These genes were sequentially named *HaPEBP1* through *HaPEBP12* according to their chromosomal positions in the T2T sunflower genome. The analysis and comparison of the CDS lengths, predicted protein sequences, MWs, isoelectric points (pI), subcellular localizations, and chromosomal distributions of the 12 genes were carried out (Supplementary Table S1). The CDS lengths ranged from 444 bp (*HaPEBP11*) to 648 bp (*HaPEBP2*), with an average length of 530 bp. Among them, *HaPEBP2* encoded the longest PEBP protein with 215 amino acids, while *HaPEBP11* encoded the shortest, comprising 147 amino acids. The MWs of these proteins ranged from 16.89 kDa to 24.50 kDa, with an average of 19.76 kDa. The pI values spanned from 4.91 (HaPEBP3) to 10 (HaPEBP8), with four HaPEBP proteins classified as acidic (pI < 7) and eight as basic (pI > 7). Further, all identified HaPEBP proteins were predicted to be hydrophilic, as evidenced by their negative Grand Average of Hydropathicity (GRAVY) values, with HaPEBP11 exhibiting the highest hydrophilicity.

**Figure 1:**
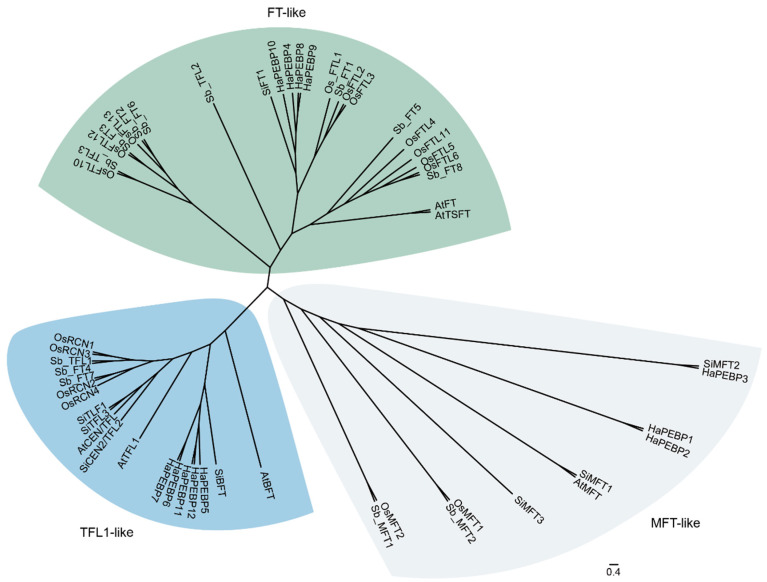
Phylogenetic tree of PEBP genes among five plants. The 68 PEBP genes, including 12 *HaPEBP* genes from sunflower (*H. annuus*), 17 *OsPEBPs* from rice (*O. sativa*), 6 *AtPEBPs* from *A. thaliana*, 8 *SiPEBPs* from sesame (*S. indicum*), and 13 *SbPEBPs* from sorghum (*S. bicolor*), are classified into three subgroups, i.e., FT-like (in green), TFL1-like (in blue), and MFT-like (in white).

To better understand the evolutionary relationships of the *HaPEBP* gene family, a phylogenetic tree was constructed using the 12 sunflower PEBP proteins alongside 17 homologs from rice, 8 from sesame, 13 from sorghum, and 6 from *A. thaliana* (Figure 1, Supplementary Table S2). The analysis classified the 68 PEBP proteins into three major subfamilies: 30 genes in the FT-like subgroup, 24 in the TFL1-like subgroup, and 14 in the MFT-like subgroup.

### 2.2. Conserved Motifs and Cis-Acting Elements of HaPEBP Genes

**Figure 2:**
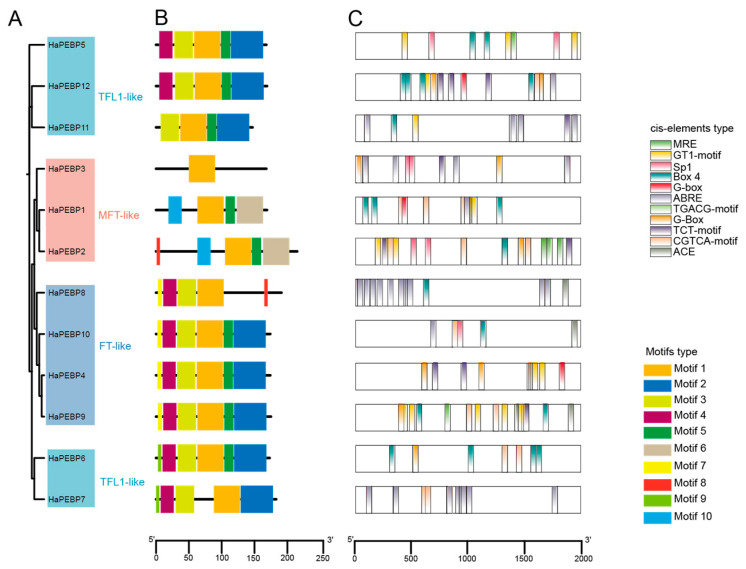
Phylogenetic and conserved motif analysis of *HaPEBP* genes. (**A**) Phylogenetic tree of the 12 HaPEBP proteins constructed using the Maximum Likelihood (ML) method. The 12 HaPEBPs are divided into four distinct groups, i.e., FT (in blue), TFL (in deep blue), MFT (in red), and BFT (in vermilion). (**B**) Distribution of conserved motifs within each HaPEBP protein. Ten distinct motifs, named Motifs 1 to 10, are shown in colorful blocks. The black scale bar at the bottom represents the distance between motifs. (**C**) Distribution of *cis*-acting elements related to light response and plant growth and development in the putative promoters of *HaPEBP* genes. A total of 31 cis-acting elements are identified in the promoters of the 12 *HaPEBP* genes. The 12 most common elements in *HaPEBP* genes are shown in different segments in the figure. The black scale indicates the sequence position of *HaPEBP* promoters.

To gain further insights into the phylogenetic relationship of *HaPEBP* genes in sunflower, a phylogenetic tree was constructed using the Maximum Likelihood (ML) method via FastTree [26]. The resulting tree topology closely mirrored the broader phylogenetic tree presented in Figure 1, except for the TFL1-like subgroup, which formed two distinct clusters (Figure 2A). The *HaPEBP* family comprised 4 FT-like, 5 TFL1-like, and 3 MFT-like genes, consistent with previous gene classification results (Supplementary Table S2).

Subsequently, an analysis of conserved motifs on the 12 HaPEBP proteins harboring the PEBP domain was performed using the MEME program (Figure 2B). This analysis identified ten distinct motifs, labeled Motif 1 to Motif 10. Motifs 1 to 6 were highly conserved across the family, with Motif 1 appearing in all 12 *HaPEBP* genes. Within the FT-like subgroup, nearly all four members possessed six motifs: *HaPEBP4*, *HaPEBP9*, and *HaPEBP10* contained Motifs 1–6, while *HaPEBP8* lacked Motifs 2 and 5 but possessed Motif 8. The TFL1-like subgroup displayed greater motif diversity, with each gene containing between four and six motifs. The MFT-like subgroup showed the highest variability: *HaPEBP1* included Motifs 1, 5, 6, and 10; *HaPEBP2* featured Motifs 1, 5, 6, 8, and 10; and *HaPEBP3* contained only Motif 1.

Given the crucial role of *cis*-acting elements in regulating gene expression, the 2000 bp upstream regions of the *HaPEBPs* were analyzed using the PlantCARE database to predict potential *cis*-acting elements (Figure 2C, Supplementary Table S3). The analysis detected 2109 occurrences of 77 distinct *cis*-acting element types across the promoter regions of 12 *HaPEBPs*. The number of *cis*-acting elements per gene ranged from 134 in *HaPEBP8* to 301 in *HaPEBP9*, with an average of 176 elements per gene. These elements were classified into seven functional categories: hormone-responsive, growth- and development-related, stress-responsive, light-responsive, site-binding, promoter-related, and other functional elements. A majority of *HaPEBP* promoters featured CAAT or TATA boxes, which are typical *cis*-acting elements in promoter and enhancer regions (Figure 2C). Furthermore, 35 distinct *cis*-acting elements, including ABRE, G-Box, and Box4, were linked to responses involving light, hormone, promoter activity, and developmental processes. The widespread distribution of these elements across *HaPEBP* promoters indicates their potential roles in diverse biological processes.

To better understand the compositions and potential functions of the 12 *HaPEBP* genes, we analyzed their gene structures, conserved sequences, and exon/intron organization (Supplementary Figure S1, Supplementary Table S1). The analysis revealed that the number of exons per gene varied from 2 to 4. Interestingly, eight *HaPEBP* genes (67%) featured four exons and three introns, while the others had fewer, implying an intricate RNA splicing process. Within the FT-like subgroup, all four genes consistently possessed four exons and three introns. Similarly, most of the five *TFL1*-like genes shared this structure, except for *HaPEBP7*, which had only two exons and one intron. In contrast, members of the MFT-like subgroup exhibited fewer exons and introns: two genes contained three exons and two introns, while *HaPEBP3* had just two exons and one intron. These observations suggest that, despite high conservation in the gene structures and protein motifs within the *HaPEBP* family, substantial divergence has occurred during evolutionary.

### 2.3. Chromosomal Localization and Gene Ontology Analysis of HaPEBP Genes

**Figure 3:**
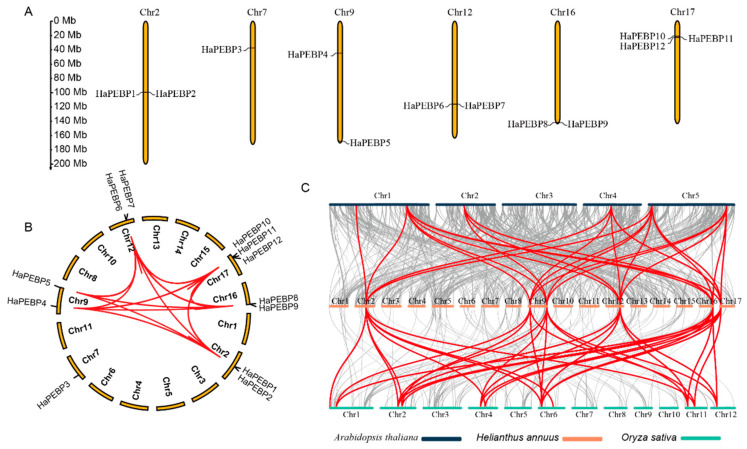
Syntenic distribution of *HaPEBP* genes in the sunflower genome. (**A**) Distribution and chromosomal localization of *HaPEBP* genes. Orange column represents sunflower chromosomes. (**B**) Synteny analysis and distribution pattern of *HaPEBP* genes. Red line indicates the syntenic gene pair within the *HaPEBP* gene family. (**C**) Collinearity analysis of *PEBP* genes among the genomes of *Arabidopsis*, sunflower, and rice. The chromosomes of Arabidopsis, sunflower, and rice are shown in black, orange, and green segments, respectively. Gray lines represent the overall gene collinearity between two crops. Red lines highlight the collinearity specific to the *PEBP* gene family.

Utilizing the T2T genome of sunflower, a chromosomal mapping and comparative analysis of *HaPEBP* genes was conducted for the first time. The analysis showed that the 12 *HaPEBP* genes were unevenly distributed across the six chromosomes, with the remaining 11 chromosomes lacking any *HaPEBP* genes (Figure 3A). Notably, chromosome 17 contained three *HaPEBP* genes, while chromosomes 2, 9, 12, and 16 each carried two *HaPEBP* genes. Only one *HaPEBP* gene was found on chromosome 7. Six *HaPEBP* genes were transcribed in the same direction as the reference genome, whereas the other six were transcribed in the opposite direction.

Subsequently, a homolog gene analysis of *HaPEBP* in sunflower was carried out using MCScanX software (Figure 3B). This analysis identified 88 homologous gene pairs within the *HaPEBP* gene family (Supplementary Table S4). Of these, nine genes were attributed to tandem duplication events. *HaPEBP4* and *HaPEBP5*, located on chromosome 9, were found to originate from dispersed duplication blocks, while *HaPEBP3* on chromosome 7 arose from a dispersed duplication event (Supplementary Table S6). These findings indicate that tandem duplication events have played a crucial role in the evolutionary expansion of the *HaPEBP* gene family in sunflower.

Additionally, a conserved collinear analysis of the *PEBP* gene family across sunflower, *Arabidopsis*, and rice was carried out (Figure 3C). Specifically, 44 orthologous gene pairs were identified between sunflower (*HaPEBPs*) and *Arabidopsis* (*AtPEBPs*), with each *AtPEBP* having two or more corresponding orthologs in the sunflower genome. Notably, *At_FT* and *At_TFL1* possessed over ten orthologous copies in sunflower (Supplementary Table S6). Furthermore, 108 orthologous gene pairs were found between sunflower (*HaPEBPs*) and rice (*OsPEBPs*), a number exceeding those found between sunflower and *Arabidopsis*. This difference is likely attributed to the higher number of *OsPEBPs* (12) compared to *AtPEBPs* (6). The identification of these orthologous genes, with potential similar functions, provided valuable insights into the characteristics of *HaPEBPs*.

### 2.4. Expression Characterization Analysis of HaPEBP Genes in Different Sunflower Tissues

**Figure 4:**
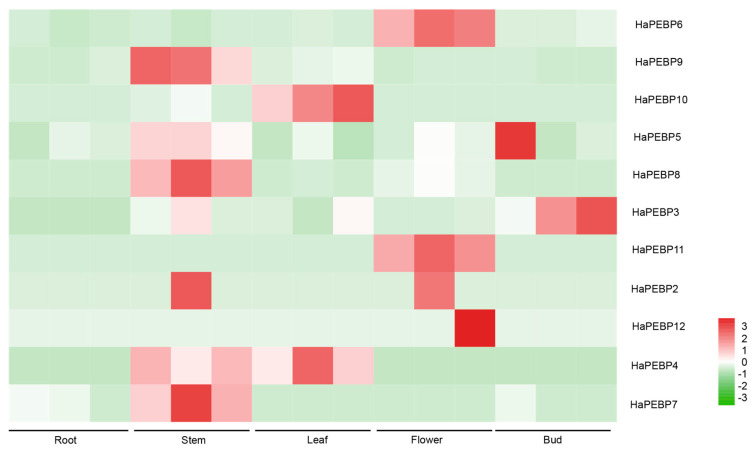
Expression profiles of the 12 *HaPEBP* genes in different tissues. Sunflower tissues, including root, stem, leaf, bud, and flower, are applied for gene expression analysis. The color scale represents the log2-transformed FPKM value. The high and the low expression levels of each gene are individually shown in dark red and bright green, respectively.

To explore the expression profiles and potential functions of *HaPEBP* genes, the transcriptome data from five different tissues (root, stem, leaf, bud, and flower) of the sunflower cv. ‘Huoli’ were analyzed (Figure 4). Out of the 12 identified *HaPEBP* genes, *HaPEBP1* was not expressed in any of the tissues examined. The other 11 genes exhibited the tissue-specific expression patterns (Figure 4). Notably, *HaPEBP6* and *HaPEBP11* demonstrated the highest expression levels in flower tissue. Additionally, five *HaPEBP* genes (*HaPEBP4*, *HaPEBP5*, *HaPEBP7*, *HaPEBP8*, and *HaPEBP9*) were predominantly expressed in the stem, while *HaPEBP3* showed elevated expression in the bud tissue, implying a potential role in regulating shoot branching.

**Figure 5:**
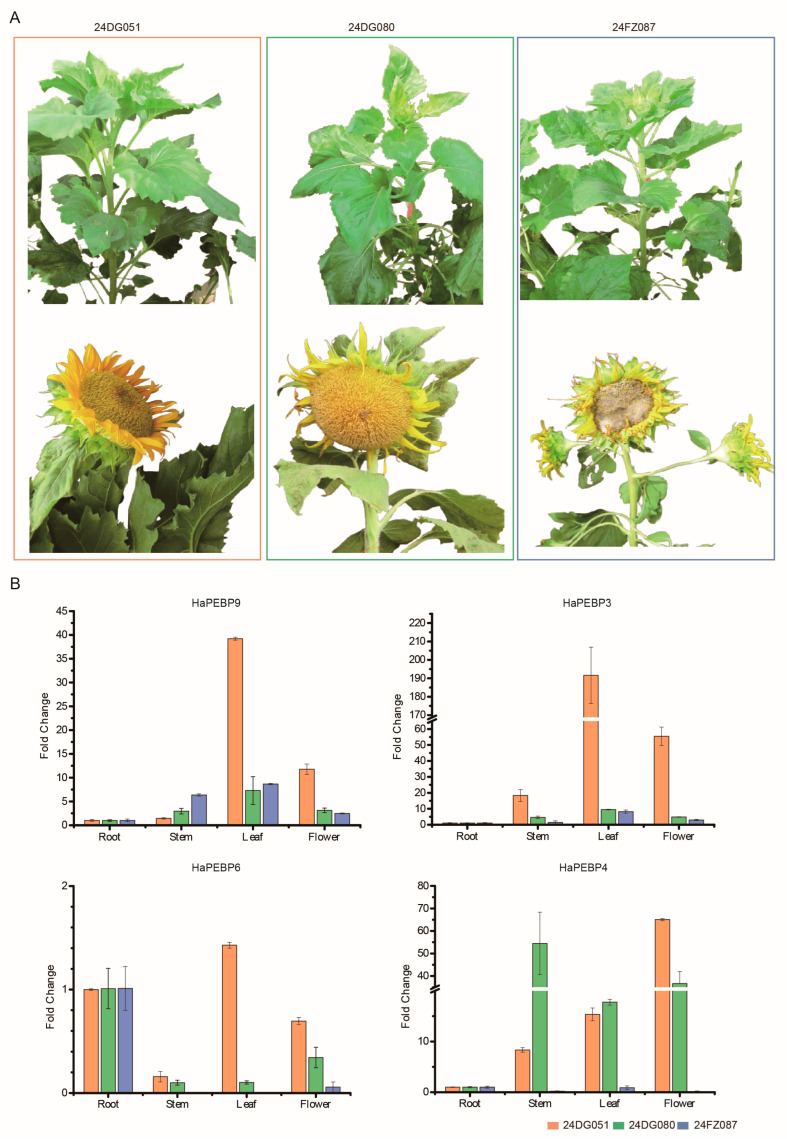
Comparison of *HaPEBP* gene expression and phenotypic characteristics across three sunflower varieties. (**A**) Phenotypic images of three sunflower germplasm accessions: 24DG051 (uniculm-type, left), 24DG080 (uniculm-type, middle), and 24FZ087 (branching-type, right). The top panel shows plant images at the pre-flowering stage, and the bottom panel displays flower images at the full-bloom stage for each variety. (**B**) Relative expression levels of four *HaPEBP* genes in the three sunflower varieties. The bar represents the mean ± standard error (SE) from three independent biological replicates. The germplasm accessions are indicated by color: 24FZ087 (branching-type) in blue, 24DG051 (uniculm-type) in orange, and 24DG080 (uniculm-type) in green.

To confirm the accuracy of the transcriptome-based expression data, four *HaPEBP* genes (*HaPEBP3*, *HaPEBP4*, *HaPEBP6*, and *HaPEBP9*) were selected for qRT-PCR analysis. Expression levels were assessed in four different tissues across both uniculm-type germplasms (24DG080 and 24DG051) and branching-type germplasm (24FZ087) (Figure 5). The qRT-PCR results aligned with the transcriptome findings, showing that *HaPEBP3* and *HaPEBP9* were highly expressed in the leaves and flowers of the uniculm-type line 24DG051. In contrast, *HaPEBP4* maintained low expression in the stem, leaves, and flowers of the branching-type line 24FZ087. Additionally, *HaPEBP6* consistently exhibited low expression in the stem, leaves, and flowers across all three sunflower materials. These findings indicate that the *HaPEBP* gene family displays tissue-specific and genotype-dependent expression patterns, suggesting a potential regulatory role in shaping plant architecture among different sunflower varieties.

### 2.5. Subcellular Localization Analysis of HaPEBPs

**Figure 6:**
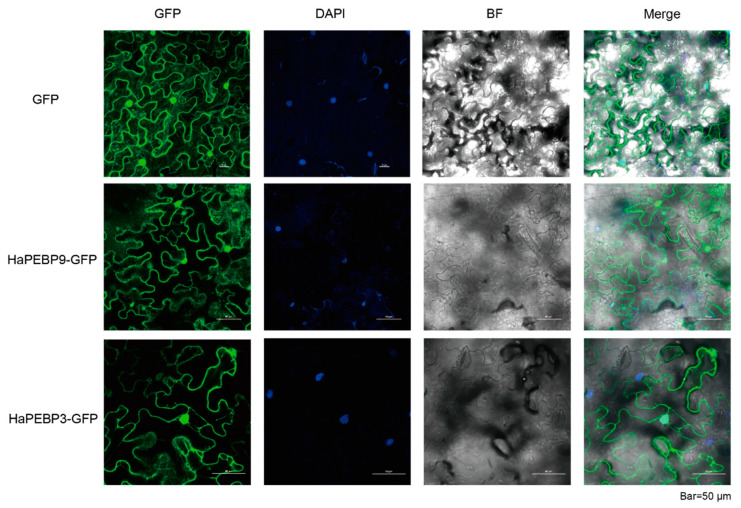
Subcellular localization of the HaPEBP-GFP fusion protein in *N. benthamiana* leaves. GFP alone is used as a control. Nuclei are counterstained with DAPI.

Subcellular localization predictions using DeepLoc 2.0 indicated that the majority of HaPEBP proteins (10 out of 12) were located in the cytoplasm. However, two proteins (HaPEBP1 and HaPEBP3) were predicted to have dual location in both the cytoplasm and nucleus (Supplementary Table S7). Subsequent experimental validation through transient expression in tobacco confirmed that HaPEBP3 and HaPEBP9 were present in both the cytoplasm and nucleus (Figure 6).

### 2.6. Three-Dimensional Structure and Protein–Protein Interaction Analysis of HaPEBPs

**Figure 7:**
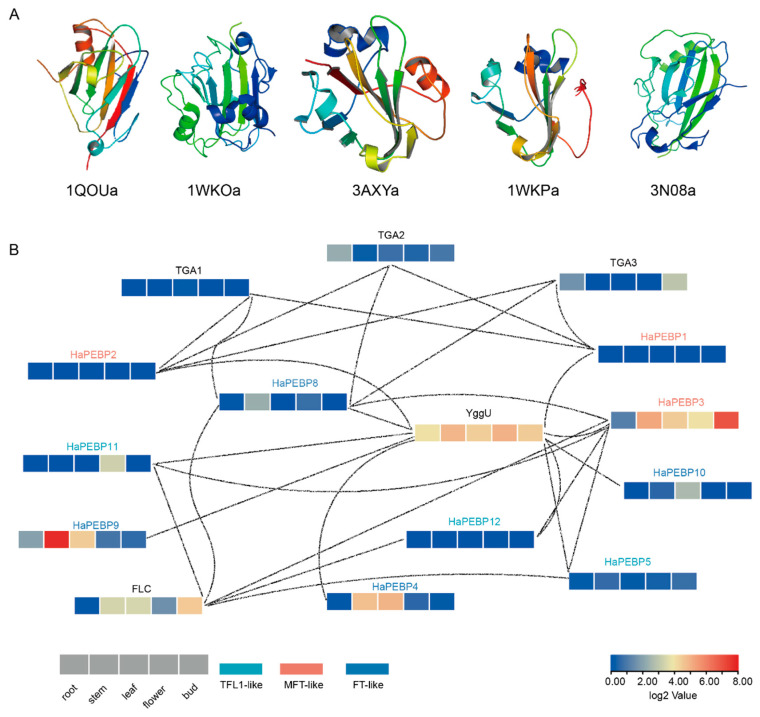
Three-dimensional structure and PPI networks of HaPEBP proteins. (**A**) Five structure models of HaPEBPs. The models are determined using RCSC PDB. (**B**) PPI analysis of HaPEBP proteins. The network is conducted using the STRING database.

To investigate the protein function of the *HaPEBP* gene family in sunflower, the three-dimensional (3D) structures of the 12 HaPEBP proteins were predicted using homology modeling based on their amino acid sequences and validated templates (Figure 7A, Supplementary Table S8). Structural analysis grouped the HaPEBP proteins into five distinct structural classes (Figure 7A), all of which shared common features such as multiple α-helices and extensive β-strands. Of these, eight proteins (67%) showed the structural similarity to the templates with PDB IDs 3AXY and 1QOU. HaPEBP1 aligned with the structure of 1WKP, HaPEBP3 exhibited similarity to 3N08, and HaPEBP6 and HaPEBP7 matched with 1WKO (Supplementary Table S8). To further explore the potential PPIs, an interaction network analysis was conducted using the STRING database (Figure 7B). The HaPEBP proteins showed extensive connections both within the family and with five other specific proteins (Figure 7B). Notably, several transcription factors (TFs), including FLOWERING LOCUS C (FLC) and TGACG-binding (TGA), exhibited differential expression across various tissues, indicating their involvement in key molecular mechanisms and biological processes related to flowering and development.

## 3. Discussion

The *PEBP* gene family plays a vital role in regulating key developmental and physiological processes in plants, including flowering time, plant architecture, floral organogenesis, seed germination, and responses to abiotic stress [2,17]. In sunflower, *FT* genes have been recognized as primary regulators of flowering time [27]. However, a comprehensive analysis of the entire *HaPEBP* gene family and its regulatory characteristics in sunflower has not yet been conducted.

In this study, 12 *HaPEBP* genes from the T2T sunflower genome were identified and characterized through combined HMM and BLAST search methods, with further validation through the PfamScan tool. All 12 *HaPEBP* genes were found to possess complete PBP domains and demonstrated evolutionary features. Expression profile analysis suggested that part of the *HaPEBP* genes might play various roles in regulating flowering time and plant architecture, offering valuable insights into the underlying molecular mechanisms governing these traits in sunflower.

### 3.1. Evolution Characteristics of HaPEBP Genes

In sunflower, the 12 identified *HaPEBP* genes display unevenly distribution across six chromosomes. Of these, nine *HaPEBP* genes exhibit evolutionary patterns primarily driven by tandem duplicated events, a common evolutionary mechanism that increases gene copy number and enables both functional diversification and sub-functionalization. Although whole-genome duplication (WGD) is prevalent in numerous plant species, our findings indicate that tandem duplications represent the primary force behind the expansion of the *PEBP* gene family in sunflower. This has led to higher numbers of *HaPEBP* genes compared to most eudicots, where this family typically contains fewer than ten genes [10].

Based on multi-alignments and phylogenetic analysis, the *HaPEBP* genes were categorized into three subgroups: TFL1-like, FT-like, and MFT-like (Figure 2A). Comparative phylogenetic analysis with other plant species, such as rice (17 *OsPEBPs*), sorghum (13 *SbPEBPs*), sesame (8 *SiPEBPs*), and *A. thaliana* (6 *AtPEBPs*), validated this classification (Figure 1), aligning with patterns observed in other dicot oilseed crops like sesame and peanut [20,28]. Notably, the classification of four sorghum *PEBP* (*SbPEBP*) genes was revised, with *Sb_TFL3* and *Sb_TFL2* being reclassified as FT-like, and *Sb_FT7* and *Sb_FT* being reassigned to the TFL-like group (Figure 1, Supplementary Table S2). Additionally, 44 orthologous gene pairs were identified between *HaPEBPs* and *AtPEBPs*, and 108 pairs between *HaPEBPs* and *OsPEBPs* (Figure 3C).

In *Arabidopsis*, the *FT* gene acts as a key regulator of flowering time by encoding a protein (florigen) that, when transported from the leaves to the shoot apex, interacts with the basic leucine zipper (bZIP) transcription factor *FD* to activate floral meristem identity genes such as *LEAFY* (*LFY*) and *APETALA1* (*AP1*), thus promoting flowering [29]. *AtFT* also interacts with the transcription factor BRANCHED1 (*BRC1*) to influence shoot branching [30]. Similarly, ectopic overexpression of pepper *CaFT5* and *CaFT6* in tobacco accelerates flowering, increases branching, and reduces leaf size, demonstrating their conserved roles in flowering promotion and plant form. *CaFT5* is mainly expressed in shoot apical meristems and flowers, whereas *CaFT6* is enriched in roots and fruits, implying redundant functions in branching and fruit development [8]. Conversely, the *TFL1* gene suppresses flowering and preserves the inflorescence meristem identity by also interacting with *FD*. Loss of *TFL1* function results in early flowering, whereas its overexpression delays flowering and inhibits terminal flower formation [1,31]. In rice, *TFL1*-related genes such as *RCN1*, *Ghd7*, and *DTH8* also inhibit flowering. *RCN1* delays flowering by competing with *Hd3a* for binding 14-3-3 proteins and *OsFD1*, while *Ghd7* and *DTH8* repress the expression of key flowering genes such as *Ehd1*, *Hd3a*, and *RFT1* under long-day conditions, thereby delaying flowering [19,32]. Moreover, CRISPR/Cas9-mediated knockouts of *FT*-like and *TFL1*-like homologs in petunia yielded more compact, highly branched plants that flowered early, underscoring the parallel functions of *PEBP* subfamily members across angiosperms [33]. These observations suggest that the *FT*-like and *TFL*-like subfamily members of *HaPEBP* genes may have conserved roles similar to their orthologous counterparts in *Arabidopsis*, rice, and petunia, particularly in regulating flowering and shaping plant form.

### 3.2. Structure Variation of HaPEBP Genes

*HaPEBP* genes within the same subfamilies exhibited similar gene structures, while notable structural variations were observed among genes from different subfamilies (Figure 2B,C). Multiple sequence alignments indicated that all members of the sunflower *PEBP* gene possess the conserved motifs ‘D-P-d-x-P’ and ‘G-x-H-R’ (Supplementary Figure S2). Interestingly, the MFT subfamily exhibited substantial polymorphism across plants, particularly among angiosperms, where the *MFT* genes have evolved into distinct subgroups, reflecting their functional diversification and evolutionary adaptation [15].

The analysis of exon–intron structure revealed that 8 out of the 12 *HaPEBP* genes typically consist of four exons and three introns, an arrangement consistent with other *PEBP* genes [3,34]. However, exceptions were observed in genes like *HaPEBP3* and *HaPEBP7*, each containing only two exons and a single intron (Supplementary Figure S1). Corresponding alterations were also noted in conserved protein motifs; for instance, *HaPEBP3* retained only one motif, a pattern also documented in pepper [8]. The consistent motif distribution within each *HaPEBP* subfamily (Figure 2B) highlights their evolutionary conservation, while the observed variations point to a balance between conservation and divergence in gene function.

*Cis*-acting regulatory elements, typically located within 2000 base pairs upstream of a gene, play a crucial role in modulating gene expression. These elements are instrumental in plant responses to various abiotic stresses and are integral to growth and developmental processes across diverse crop species [35]. In the putative promoters of *PEBP* genes, *cis*-acting elements have been shown to mediate responses to abiotic and biotic stresses, regulate hormone-responsive transcription, and coordinate key processes in plant growth and development [7]. Typical light response elements, such as GT1-motif, Box 4, G-box, and TCT-motif, which could be analyzed similarly to the *FT* expression assessment in pears and *Dendrobium* species [36,37]. These elements mediate the response to light, influencing flowering time and photoperiod sensitivity. Additionally, the various *HaPEBP* members exhibit characteristics associated with different plant hormones, including ABRE for abscisic acid (ABA), AuxRR-core and TGA-element for auxin, CGTCA-motif for methyl jasmonate (MeJA), GARE-motif and TATC-box for gibberellin, and TCA-element for salicylic acid (SA). These findings suggest that *HaPEBP* genes likely contribute to the regulation of plant hormone responses.

Collinearity analysis within sunflower revealed 88 intra-species gene pairs, with the majority of *HaPEBP* genes demonstrating clear evidence of tandem duplication (Figure 3B, Supplementary Table S5). The analysis of Ka/Ks ratios for all *HaPEBP* genes consistently produced values less than 1 (Supplementary Table S9), suggesting that purifying selection has preserved both the structure and function of this gene family. Although tandem duplication, WGD, and segmental duplication are all established drivers of plant genome evolution [38,39], the findings indicate that tandem duplication is the primary mechanism driving the expansion and diversification of the *HaPEBP* gene family in sunflower.

### 3.3. Biological Functions of HaPEBP Genes

Several studies have shown that the expression patterns of *PEBP* genes are closely associated with their biological functions. For instance, in *A. thaliana*, the *FT* gene (*AtFT*) is expressed in both reproductive and vegetative organs, where it promotes flowering in conjunction with flowering-related *LFY* gene [40]. Similarly, this study found that *HaPEBP* genes exhibited tissue-specific expression, indicating their involvement in various aspects of growth and development. Notably, *HaPEBP9* and *HaPEBP10*, both members of the *FT*-like subfamily, were predominantly expressed in leaves (Figure 4). Their promoter regions contained an abundance of light-responsive elements, including Sp1, G-box, and Box-4, suggesting that these genes may be light-sensitive and play significant roles in controlling flowering time [41,42]. Another *FT*-like gene, *HaPEBP8*, showed predominant expression in stems, indicating its potentially important role in developmental regulation. These *FT*-like genes were expressed across a range of tissues, and their response elements reflected this diversity (Figure 3C). This widespread tissue expression suggests that the *HaPEBP* genes have multifunctional roles in sunflower growth and development. Similar to *AtFT*, which encodes florigen proteins essential for reproductive transition and plant architecture, the homologous *HaPEBP* genes likely participate in a comparable regulatory process. Phylogenetic analysis revealed that the three *FT*-like genes shared a high degree of amino acid similarity with *AtFT* [43,44], with only minimal differences (Figure 1). Furthermore, the three HaPEBP proteins were localized in the cytoplasm, a characteristic also observed in *AtFT* and the cotton homologous proteins GhFT [45].

Plant architecture is a critical trait that significantly influences both the development of terminal flowers and the costs of sunflower production [23]. The *TFL1*-like gene, in particular, plays a crucial role in regulating the transition from vegetative to reproductive growth, thereby determining whether plants exhibit indeterminate or determinate growth habits [46]. For example, in *Arabidopsis*, *TFL1* sustains indeterminate growth by suppressing genes responsible for floral meristem identity, and mutations in the *TFL1*-like gene lead to early flowering and a switch to determinate growth [1]. A similar function is noted in sesame, where the *TFL1*-like subfamily gene *SiCEN2* influences plant form and inflorescence development. Variations in *SiCNE2*, such as LTR insertions, significantly impact branching and the timing of flowering [20]. In other crops like pigeon peas and rapeseed, mutations in *TFL1*-like homologs are associated with variations in branching, plant height, and flowering time, emphasizing the roles of *TFL1*-like genes in shaping growth habits [47,48]. Moreover, gene duplications and the subsequent functional divergence within the *TFL1*-like gene family help drive the evolution of plant architectures by modulating flowering and growth in response to environmental cues. In the present study, phylogenetic analysis revealed that five *HaPEBP* genes (*HaPEBP5*, *HaPEBP6*, *HaPEBP7*, *HaPEBP11*, and *HaPEBP12*) belonged to the *TFL1*-like subfamily and clustered closely with established regulators such as *SiCEN2* and *ATCEN* (Figure 1), suggesting that they may play similar roles in shaping plant architecture in sunflower. Transcriptome analysis further substantiated this observation, as these genes were highly expressed in key tissues, namely the stem, flower, and bud. Specifically, *HaPEBP3* demonstrated elevated expression in the stem and bud, aligning with previous studies highlighting its regulatory role in plant architecture [49]. Collectively, these findings offer valuable insights into the genetic regulation of plant architecture and denote candidate target genes for sunflower breeding efforts focused on optimizing plant architecture.

This article examined the 3D structure of HaPEBP proteins to gain deeper insights into the structural features and potential functions of the HaPEBP proteins in sunflower. Based on data from the RCSC PDB database, five comparative homology models for 12 HaPEBP proteins were available. The sequence identity ranged from 35% to 81% (Supplementary Table S8). All five models exhibited characteristic PEBP structural elements, such as multiple α-helices and a prominent β-sheet (Figure 7A). Notably, 67% of HaPEBP proteins showed structural similarity to templates with PDB IDs 3AXY and 1QOU, which correspond to PEBP protein from rice (OsFD3) and *Antirrhinum*, respectively [50,51]. Specific alignments were also observed: HaPEBP1 aligned with portions of the structure represented by PDB ID 1WKP, HaPEBP3 with PDB ID 3N08, and both HaPEBP6 and HaPEBP7 with PDB ID 1WKO [52,53]. These structural similarities indicate a conserved fold among HaPEBPs, likely critical for their function. This conserved structure suggests a preserved molecular mechanism underlying their biological functions and highlights their potential involvement in key regulatory pathways across plant species.

The PPI network analysis of HaPEBP proteins in sunflower, conducted using the STRING database, demonstrates the high conservation and central regulatory roles of PEBP proteins in plant development (Figure 7B). A confidence score threshold of 0.4 was used to ensure reliable interactions among the proteins. Within this extensively connected network, YggU, a plant cysteine protease, emerges as a notable interactor. Cysteine proteases play multifaceted roles in plants, including response to pathogens, growth, development, senescence, programmed cell death, and the accumulation and mobilization of storage proteins, as well as responses to biotic and abiotic stresses [54]. The elevated expression of YggU across various sunflower tissues indicates its substantial role in these physiological processes. Another significant interactor is the FLC protein, a MIKC-type MADS transcription factor, that interacts with *FT* and other *PEBP* family members. *FLC* is essential for regulating flowering time and plant development by suppressing flowering integrator genes, thus controlling the transition from vegetative to reproductive stages [55]. Additionally, TGA transcription factors, part of the bZIP family, play vital roles in plant immune responses and developmental processes. Their interactions with PEBP family proteins facilitate the integration of signals from diverse pathways, enabling precise regulation of plant growth, development, and flowering. For instance, *TGA7* regulates flowering time by modulating flowering repressor genes, including *FLC* [56]. Furthermore, TGA transcription factors influence plant flowering time and organ development through interactions with PEBP family proteins [57]. The different expression levels of these transcription factors and their interactions with HaPEBP proteins reflect a complex regulatory network governing flowering and developmental processes in sunflower. Future investigation into the key *HaPEBP* genes in this interaction network may offer deeper insights into their specific roles and regulatory pathways. This study will enhance our understanding of *PEBP* gene functions in sunflower and other crops, offering potential strategies for improving plant architecture and yield through targeted breeding.

## 4. Methods and Materials

### 4.1. Plant Materials

This study utilized three sunflower germplasm accessions for gene expression analysis: 24DG080 and 24DG051 (uniculm-type), and 24FZ087 (branching-type). All materials were cultured at the Yuanyang experimental station (113°97′ E and 35°05′ N), where their phenotypes were continuously monitored and samples collected during the 2023–2024 growing season. The sunflower materials were supplied by the Economic Crop Research Institute of Henan Academy of Agricultural Sciences (ECRI-HAAS), Zhengzhou, Henan, China. Seeds were sown in the field on 7 June 2024, following standard agronomic practices. Tissue samples, including root, stem, leaf, and flower, were collected at the onset of flowering (19 July 2024), immediately frozen in liquid nitrogen, and stored at −80 °C until further analysis.

### 4.2. Identification of the PEBP Gene Family in Sunflower

This study employed the first assembled T2T genome of sunflower (Chinese variety, Sandaomei), which is publicly available in the National Genomics Data Center (NGDC) database (project no. PRJCA028800). *PEBP* protein sequences from *Arabidopsis thaliana*, retrieved from The Arabidopsis Information Resource (TAIR) database, served as queries in a BLASTP search against the sunflower protein sequences, using an E-value threshold of <1 × 10^−5^ and default parameters. Additionally, the seed file for the *PEBP* domain (PF01161) was acquired from the Protein Families Database (Pfam, v35.0) [58]. The HMMER software (v3.3.2) was employed to identify *PEBP* domains within the sunflower protein sequences [59]. Candidate genes were further validated for the presence of the *PEBP* domain using PfamScan (v1.6) [60], and only those containing a complete conserved *PEBP* domain were retained for further analysis.

### 4.3. Multiple Sequence Alignment and Phylogenetic Analysis of HaPEBP

Before exploring the diversity of HaPEBPs, full-length amino acid sequences of PBEPs from *Oryza sativa*, *A. thaliana*, *Sesamum indicum*, and *Sorghum bicolor* were retrieved from previous studies [6,7,20,34]. These sequences were aligned using MAFFT (v7.505) [61], and a phylogenetic tree was constructed using the Maximum Likelihood (ML) method in FastTree v2.1.1. The analysis employed the Jones–Taylor–Thornton (JTT) amino acid substitution model, the default setting in FastTree for protein sequence alignments, selected for its effectiveness in analyzing moderately divergent protein sequences. Tree reliability was assessed through 1000 bootstrap replications [26]. The phylogenetic tree for *HaPEBPs* was visualized using FigTree (v1.4.4, available online: http://tree.bio.ed.ac.uk/software/figtree/ (accessed on 20 June 2021).

### 4.4. Motifs and Gene Structure Analysis

The conserved motifs within each *HaPEBP* member were analyzed using the Multiple Expectation Maximization for Motif Elicitation (MEME) Suite (version 5.5.7), with parameters configured to identify a maximum of 10 motifs [62]. The gene structures of *HaPEBPs* were analyzed via the online Gene Structure Display Server (GSDS 2.0) [63].

### 4.5. Identification and Analysis of Cis-Acting Elements in Gene Promoters

The 2000 bp upstream sequence of the *HaPEBP* genes was extracted as the putative promoter regions and analyzed for *cis*-acting elements using the default settings in the PlantCARE database [64]. The results were then visualized using TBtools (version 2.210) [65].

### 4.6. Characterization, Chromosomal Localization, and Synteny Analysis of HaPEBP Genes

The genomic positions of *HaPEBP* genes were mapped using the MapGene2Chrom (v2.1) platform [66]. The sequence length, molecular weight (MW), and isoelectric point (pI) were calculated using tools available on the ExPasy website [67]. Pairwise genome comparisons among *A. thaliana*, *O. sativa*, and sunflower were performed using the JCVI software v1.4.24 [68]. Synteny regions and chromosome distributions were analyzed using MCScanX v1.0.0 with default parameters, and the results were visualized using Circos v0.52 [69].

### 4.7. Analysis of Selective Pressures on HaPEBP Genes

Homology alignment of HaPEBP protein sequences was first carried out and subsequently translated the resulting alignment into a codon-level alignment. Pairwise nonsynonymous (Ka) and synonymous (Ks) substitution rates were then calculated using KaKs_Calculator (V3.0) [70], which employs model selection and averaging methods to estimate these rates. The Ka/Ks ratio was analyzed to evaluate the selective pressures on gene duplication events among homologous *HaPEBP* genes in sunflower.

### 4.8. Subcellular Localization

The subcellular localization of *HaPEBP* proteins was predicted using the DeepLoc 2.0 online tool [71]. The coding sequence (CDS) of the target gene (without the stop codon) was amplified from sunflower materials and ligated to the N-terminus of GFP within the pCambia1300 vector to construct the *35S:Gene-GFP* recombinant expression vector. The constructed plasmids were transformed into the *Agrobacterium tumefaciens* GV3101 strain and transiently expressed in *Nicotiana benthamiana* leaves via the infiltration method. Following 8 h of incubation in the dark, the plants were moved to normal light conditions for an additional 48 h. The GFP fusion protein fluorescence signal was detected and imaged using a Nikon laser scanning confocal microscope (NiKon-AX, NIS-Elements 5.4, Tokyo, Japan). Primers used for amplification are listed in the Supplementary Table S10.

### 4.9. Expression Profile Assay of HaPEBP Gene and RNA-Seq Data Analysis

To investigate the expression patterns of *HaPEBP* genes across different sunflower tissues, the Fragments Per Kilobase of exon model per Million mapped reads (FPKM) matrix data for the sunflower cv. ‘Huoli’ were obtained from the NCBI database (accession number GSE221055) [49]. The log2 fold change (log2FC) for each treatment was calculated and visualized as heatmaps using the R package ‘pheatmap’ (version 1.0.12).

Total RNA was isolated from various sunflower tissues (root, stem, leaf, bud, and seed) across three germplasm accessions, using the Polysaccharide Polyphenol Total RNA Kit (Tiangen, Beijing, China). The quality of the RNA samples was assessed using a spectrophotometer (Nanodrop, Thermo Scientific, Waltham, MA, USA) and by performing agarose gel electrophoresis. cDNA synthesis was carried out using DNase I (Thermo Scientific, #EN0521) and the RevertAid™ First Strand cDNA Synthesis Kit (Thermo Scientific, #K1622). The *β-tubulin* gene served as the internal reference gene for quantitative real-time PCR (qRT-PCR) analysis. The relative expression levels of *HaPEBP* genes were calculated using the 2^−ΔΔCt^ method [72]. qRT-PCR was conducted using the Roche LightCycler^®^ 480 II Real-Time PCR System (Roche Diagnostics, Basel, Switzerland). Primer pairs specific to *HaPEBP* alleles (Supplementary Table S10) were designed using Primer Premier 5.0 (available online: http://www.premierbiosoft.com/primerdesign/index.html (accessed on 8 January 2021). All reactions were performed in triplicate, and the gene expression levels were normalized against the *β*-tubulin gene.

### 4.10. Analysis of the Three-Dimensional Structure and Protein–Protein Interactions of HaPEBP Proteins

To investigate the three-dimensional (3D) structure of HaPEBP proteins, comparative modeling was performed using the RCSB Protein Data Bank (PDB) [73]. The resulting 3D structures were visualized and examined using PyMOL (v2.5.4) [74]. PPIs were predicted using the STRING database v10 [75], employing an interaction score threshold of 0.4 to ensure high confidence in the predicted interactions. The resulting PPI networks were then visualized and analyzed using Cytoscape (v3.9.1) [76].

## 5. Conclusions

In this study, we identified 12 *HaPEBP* genes within the sunflower T2T genome and performed a comprehensive analysis of their chromosomal distribution, phylogenetic relationships, gene structures, and expression profiles. These genes were categorized into three distinct subfamilies: TFL1-like, FT-like, and MFT-like, each characterized by conserved sequence motifs and largely influenced by tandem duplication events. The analysis of the promoter regions of 12 *HaPEBP* genes revealed 77 distinct types of cis-acting elements, with 35 elements potentially associated with light responsiveness and plant growth and development. Expression profiling based on RNA-seq and RT-PCR indicated that *HaPEBP* genes exhibit tissue-specific expression patterns. PPI network analysis further confirmed their central roles in regulating flowering and plant architecture. Collectively, these findings shed light on the structure, evolution, and functional diversification of the *HaPEBP* gene family and provide valuable genetic resources for the development of sunflower cultivars with adaptive flowering time and optimized plant architecture.

## Data Availability

The original contributions presented in this study are in the article/Supplementary Materials, and further inquiries can be directed to the corresponding authors.

## Supplementary Materials

The following supporting information can be downloaded at https://www.mdpi.com/article/10.3390/ijms26104602/s1.

## References

[1] Wickland D.P., Hanzawa Y. (2015). The *FLOWERING LOCUS T*/*TERMINAL FLOWER 1* gene family: Functional evolution and molecular mechanisms. Mol. Plant.

[2] Trakul N., Rosner M.R. (2005). Modulation of the MAP kinase signaling cascade by Raf kinase inhibitory protein. Cell Res..

[3] Karlgren A., Gyllenstrand N., Källman T., Sundström J.F., Moore D., Lascoux M., Lagercrantz U. (2011). Evolution of the PEBP gene family in plants: Functional diversification in seed plant evolution. Plant Physiol..

[4] Kikuchi R., Kawahigashi H., Ando T., Tonooka T., Handa H. (2009). Molecular and functional characterization of PEBP genes in barley reveal the diversification of their roles in flowering. Plant Physiol..

[5] Sun Y., Jia X., Yang Z., Fu Q., Yang H., Xu X. (2023). Genome-wide identification of PEBP gene family in *Solanum lycopersicum*. Int. J. Mol. Sci..

[6] Wang Z., Zhou Z., Liu Y., Liu T., Li Q., Ji Y., Li C., Fang C., Wang M., Wu M. (2015). Functional evolution of phosphatidylethanolamine binding proteins in soybean and *Arabidopsis*. Plant Cell.

[7] Zhao C., Zhu M., Guo Y., Sun J., Ma W., Wang X. (2022). Genomic survey of *PEBP* gene family in rice: Identification, phylogenetic analysis, and expression profiles in organs and under abiotic stresses. Plants.

[8] Wu X., Gan Z., Xu F., Qian J., Qian M., Ai H., Feng T., Lu X., Li R., Huang X. (2024). Molecular characterization of pepper *PEBP* genes reveals the diverse functions of *CaFTs* in flowering and plant architecture. Sci. Hortic..

[9] Putterill J., Varkonyi-Gasic E. (2016). FT and florigen long-distance flowering control in plants. Curr. Opin. Plant Biol..

[10] Liu Y.Y., Yang K.Z., Wei X.X., Wang X.Q. (2016). Revisiting the phosphatidylethanolamine-binding protein (PEBP) gene family reveals cryptic *FLOWERING LOCUS T* gene homologs in gymnosperms and sheds new light on functional evolution. New Phytol..

[11] Yamaguchi A., Kobayashi Y., Goto K., Abe M., Araki T. (2005). *TWIN SISTER OF FT* (*TSF*) Acts as a Floral Pathway Integrator Redundantly with *FT*. Plant Cell Physiol..

[12] Yoo S.J., Chung K.S., Jung S.H., Yoo S.Y., Lee J.S., Ahn J.H. (2010). *BROTHER OF FT AND TFL1* (*BFT*) has *TFL1*-like activity and functions redundantly with *TFL1* in inflorescence meristem development in *Arabidopsis*. Plant J..

[13] Ryu J.Y., Lee H.-J., Seo P.J., Jung J.-H., Ahn J.H., Park C.-M. (2014). The *Arabidopsis* Floral Repressor BFT Delays Flowering by Competing with FT for FD Binding under High Salinity. Mol. Plant.

[14] Cai Z., Xian P., Cheng Y., Zhong Y., Yang Y., Zhou Q., Lian T., Ma Q., Nian H., Ge L. (2023). MOTHER-OF-FT-AND-TFL1 regulates the seed oil and protein content in soybean. New Phytol..

[15] Hedman H., Källman T., Lagercrantz U. (2009). Early evolution of the MFT-like gene family in plants. Plant Mol. Biol..

[16] Zhu Y., Klasfeld S., Jeong C.W., Jin R., Goto K., Yamaguchi N., Wagner D. (2020). TERMINAL FLOWER 1-FD complex target genes and competition with FLOWERING LOCUS T. Nat. Commun..

[17] Su T., Wu Y., Fang C., Liu B., Lu S., Kong F., Liu H. (2024). The Critical Roles of Phosphatidylethanolamine-Binding Proteins in Legumes. Plant Cell Environ..

[18] Zhao H., Huang X., Yang Z., Li F., Ge X. (2023). Synergistic optimization of crops by combining early maturation with other agronomic traits. Trends Plant Sci..

[19] Tamaki S., Matsuo S., Wong H.L., Yokoi S., Shimamoto K. (2007). Hd3a protein is a mobile flowering signal in rice. Science.

[20] Miao H., Wang L., Qu L., Liu H., Sun Y., Le M., Wang Q., Wei S., Zheng Y., Lin W. (2024). Genomic evolution and insights into agronomic trait innovations of *Sesamum* species. Plant Commun..

[21] Mandel J.R., Dikow R.B., Siniscalchi C.M., Thapa R., Watson L.E., Funk V.A. (2019). A fully resolved backbone phylogeny reveals numerous dispersals and explosive diversifications throughout the history of Asteraceae. Proc. Natl. Acad. Sci. USA.

[22] Dogra S., Gupta A.K., Kumar V., Naik B., Mishra P. (2024). Chapter 7—*Helianthus annuus* L.. Edible Flowers.

[23] Puttha R., Venkatachalam K., Hanpakdeesakul S., Wongsa J., Parametthanuwat T., Srean P., Pakeechai K., Charoenphun N. (2023). Exploring the Potential of Sunflowers: Agronomy, Applications, and Opportunities within Bio-Circular-Green Economy. Horticulturae.

[24] Blackman B.K. (2013). Interacting duplications, fluctuating selection, and convergence: The complex dynamics of flowering time evolution during sunflower domestication. J. Exp. Bot..

[25] Blackman B.K., Michaels S.D., Rieseberg L.H. (2011). Connecting the sun to flowering in sunflower adaptation. Mol. Ecol..

[26] Price M.N., Dehal P.S., Arkin A.P. (2010). FastTree 2–approximately maximum-likelihood trees for large alignments. PLoS ONE.

[27] Zheng T., Li P., Li L., Zhang Q. (2021). Research advances in and prospects of ornamental plant genomics. Hortic. Res..

[28] Jin H., Tang X., Xing M., Zhu H., Sui J., Cai C., Li S. (2019). Molecular and transcriptional characterization of phosphatidyl ethanolamine-binding proteins in wild peanuts *Arachis duranensis* and *Arachis ipaensis*. BMC Plant Biol..

[29] Martignago D., da Silveira Falavigna V., Lombardi A., Gao H., Korwin Kurkowski P., Galbiati M., Tonelli C., Coupland G., Conti L. (2023). The bZIP transcription factor AREB3 mediates FT signalling and floral transition at the *Arabidopsis* shoot apical meristem. PLoS Genet..

[30] Niwa M., Daimon Y., Kurotani K.-I., Higo A., Pruneda-Paz J.L., Breton G., Mitsuda N., Kay S.A., Ohme-Takagi M., Endo M. (2013). BRANCHED1 Interacts with FLOWERING LOCUS T to Repress the Floral Transition of the Axillary Meristems in *Arabidopsis*. Plant Cell.

[31] Jaeger K.E., Pullen N., Lamzin S., Morris R.J., Wigge P.A. (2013). Interlocking feedback loops govern the dynamic behavior of the floral transition in *Arabidopsis*. Plant Cell.

[32] Kaneko-Suzuki M., Kurihara-Ishikawa R., Okushita-Terakawa C., Kojima C., Nagano-Fujiwara M., Ohki I., Tsuji H., Shimamoto K., Taoka K.-I. (2018). TFL1-Like Proteins in Rice Antagonize Rice FT-Like Protein in Inflorescence Development by Competition for Complex Formation with 14-3-3 and FD. Plant Cell Physiol..

[33] Abdulla M.F., Mostafa K., Kavas M. (2024). CRISPR/Cas9-mediated mutagenesis of *FT*/*TFL1* in petunia improves plant architecture and early flowering. Plant Mol. Biol..

[34] Chardon F., Damerval C. (2005). Phylogenomic analysis of the PEBP gene family in cereals. J. Mol. Evol..

[35] Marand A.P., Eveland A.L., Kaufmann K., Springer N.M. (2023). *cis*-Regulatory Elements in Plant Development, Adaptation, and Evolution. Annu. Rev. Plant Biol..

[36] Zhao S., Wei Y., Pang H., Xu J., Li Y., Zhang H., Zhang J., Zhang Y. (2020). Genome-wide identification of the *PEBP* genes in pears and the putative role of *PbFT* in flower bud differentiation. PeerJ.

[37] Zhang M.-M., Zhao X., He X., Zheng Q., Huang Y., Li Y., Ke S., Liu Z.-J., Lan S. (2023). Genome-wide identification of PEBP gene family in two *Dendrobium* species and expression patterns in *Dendrobium chrysotoxum*. Int. J. Mol. Sci..

[38] Panchy N., Lehti-Shiu M., Shiu S.-H. (2016). Evolution of gene duplication in plants. Plant Physiol..

[39] Rodgers-Melnick E., Mane S.P., Dharmawardhana P., Slavov G.T., Crasta O.R., Strauss S.H., Brunner A.M., DiFazio S.P. (2012). Contrasting patterns of evolution following whole genome versus tandem duplication events in *Populus*. Genome Res..

[40] Kobayashi Y., Kaya H., Goto K., Iwabuchi M., Araki T. (1999). A pair of related genes with antagonistic roles in mediating flowering signals. Science.

[41] Borello U., Ceccarelli E., Giuliano G. (1993). Constitutive, light-responsive and circadian clock-responsive factors compete for the different I box elements in plant light-regulated promoters. Plant J..

[42] Kumar G.M., Mamidala P., Podile A.R. (2009). Regulation of Polygalacturonase-inhibitory proteins in plants is highly dependent on stress and light responsive elements. Plant Omics.

[43] Lifschitz E., Eshed Y. (2006). Universal florigenic signals triggered by *FT* homologues regulate growth and flowering cycles in perennial day-neutral tomato. J. Exp. Bot..

[44] Corbesier L., Vincent C., Jang S., Fornara F., Fan Q., Searle I., Giakountis A., Farrona S., Gissot L., Turnbull C. (2007). FT protein movement contributes to long-distance signaling in floral induction of *Arabidopsis*. Science.

[45] Li C., Zhang Y., Zhang K., Guo D., Cui B., Wang X., Huang X. (2015). Promoting flowering, lateral shoot outgrowth, leaf development, and flower abscission in tobacco plants overexpressing cotton *FLOWERING LOCUS T* (*FT*)-like gene *GhFT1*. Front. Plant Sci..

[46] Moraes T.S., Dornelas M.C., Martinelli A.P. (2019). FT/TFL1: Calibrating plant architecture. Front. Plant Sci..

[47] Mendapara I., Modha K., Patel S., Parekh V., Patel R., Chauhan D., Bardhan K., Siddiqui M.H., Alamri S., Rahman M.A. (2023). Characterization of *CcTFL1* governing plant architecture in pigeon pea (*Cajanus cajan* (L.) Millsp.). Plants.

[48] Sriboon S., Li H., Guo C., Senkhamwong T., Dai C., Liu K. (2020). Knock-out of *TERMINAL FLOWER 1* genes altered flowering time and plant architecture in *Brassica napus*. BMC Genet..

[49] Wu Y., Zhang J., Li C., Deng X., Wang T., Dong L. (2023). Genome-wide analysis of TCP transcription factor family in sunflower and identification of *HaTCP1* involved in the regulation of shoot branching. BMC Plant Biol..

[50] Taoka K.-i., Ohki I., Tsuji H., Furuita K., Hayashi K., Yanase T., Yamaguchi M., Nakashima C., Purwestri Y.A., Tamaki S. (2011). 14-3-3 proteins act as intracellular receptors for rice Hd3a florigen. Nature.

[51] Banfield M.J., Brady R.L. (2000). The structure of *Antirrhinum* centroradialis protein (CEN) suggests a role as a kinase regulator. J. Mol. Biol..

[52] Ho W.W.H., Weigel D. (2014). Structural Features Determining Flower-Promoting Activity of *Arabidopsis* FLOWERING LOCUS T. Plant Cell.

[53] Ahn J.H., Miller D., Winter V.J., Banfield M.J., Lee J.H., Yoo S.Y., Henz S.R., Brady R.L., Weigel D. (2006). A divergent external loop confers antagonistic activity on floral regulators FT and TFL1. EMBO J..

[54] Baek K.H., Choi D.I. (2008). Roles of plant proteases in pathogen defense. Plant Pathol. J..

[55] Li D., Shao L., Zhang J., Wang X., Zhang D., Horvath D.P., Zhang L., Zhang J., Xia Y. (2022). MADS-box transcription factors determine the duration of temporary winter dormancy in closely related evergreen and deciduous *Iris* spp.. J. Exp. Bot..

[56] Xu X., Xu J., Yuan C., Hu Y., Liu Q., Chen Q., Zhang P., Shi N., Qin C. (2021). Characterization of genes associated with *TGA7* during the floral transition. BMC Plant Biol..

[57] Koornneef M., Alonso-Blanco C., Vreugdenhil D. (2004). Naturally occurring genetic variation in *Arabidopsis thaliana*. Annu. Rev. Plant Biol..

[58] Mistry J., Chuguransky S., Williams L., Qureshi M., Salazar G.A., Sonnhammer E.L., Tosatto S.C., Paladin L., Raj S., Richardson L.J. (2021). Pfam: The protein families database in 2021. Nucleic Acids Res..

[59] Prakash A., Jeffryes M., Bateman A., Finn R.D. (2017). The HMMER web server for protein sequence similarity search. Curr. Protoc. Bioinform..

[60] Mistry J., Bateman A., Finn R.D. (2007). Predicting active site residue annotations in the Pfam database. BMC Bioinform..

[61] Katoh K., Standley D.M. (2013). MAFFT multiple sequence alignment software version 7: Improvements in performance and usability. Mol. Biol. Evol..

[62] Bailey T.L., Williams N., Misleh C., Li W.W. (2006). MEME: Discovering and analyzing DNA and protein sequence motifs. Nucleic Acids Res..

[63] Hu B., Jin J., Guo A.-Y., Zhang H., Luo J., Gao G. (2015). GSDS 2.0: An upgraded gene feature visualization server. Bioinformatics.

[64] Lescot M., Déhais P., Thijs G., Marchal K., Moreau Y., Van de Peer Y., Rouzé P., Rombauts S. (2002). PlantCARE, a database of plant cis-acting regulatory elements and a portal to tools for in silico analysis of promoter sequences. Nucleic Acids Res..

[65] Chen C., Wu Y., Li J., Wang X., Zeng Z., Xu J., Liu Y., Feng J., Chen H., He Y. (2023). TBtools-II: A “one for all, all for one” bioinformatics platform for biological big-data mining. Mol. Plant.

[66] Chao J.-T., Kong Y.-Z., Wang Q., Sun Y.-H., Gong D.-P., Lv J., Liu G.-S. (2015). MapGene2Chrom, a tool to draw gene physical map based on Perl and SVG languages. Yi Chuan Hered..

[67] Duvaud S., Gabella C., Lisacek F., Stockinger H., Ioannidis V., Durinx C. (2021). Expasy, the Swiss Bioinformatics Resource Portal, as designed by its users. Nucleic Acids Res..

[68] Tang H., Krishnakumar V., Zeng X., Xu Z., Taranto A., Lomas J.S., Zhang Y., Huang Y., Wang Y., Yim W.C. (2024). JCVI: A versatile toolkit for comparative genomics analysis. iMeta.

[69] Wang Y., Tang H., Wang X., Sun Y., Joseph P.V., Paterson A.H. (2024). Detection of colinear blocks and synteny and evolutionary analyses based on utilization of MCScanX. Nat. Protoc..

[70] Zhang Z. (2022). KaKs_Calculator 3.0: Calculating selective pressure on coding and non-coding sequences. Genom. Proteom. Bioinform..

[71] Thumuluri V., Almagro Armenteros J.J., Johansen A.R., Nielsen H., Winther O. (2022). DeepLoc 2.0: Multi-label subcellular localization prediction using protein language models. Nucleic Acids Res..

[72] Livak K.J., Schmittgen T.D. (2001). Analysis of relative gene expression data using real-time quantitative PCR and the 2^−ΔΔCT^ method. Methods.

[73] Burley S.K., Bhikadiya C., Bi C., Bittrich S., Chao H., Chen L., Craig P.A., Crichlow G.V., Dalenberg K., Duarte J.M. (2023). RCSB Protein Data Bank (RCSB. org): Delivery of experimentally-determined PDB structures alongside one million computed structure models of proteins from artificial intelligence/machine learning. Nucleic Acids Res..

[74] Yuan S., Chan H.S., Hu Z. (2017). Using PyMOL as a platform for computational drug design. Wiley Interdiscip. Rev. Comput. Mol. Sci..

[75] Szklarczyk D., Franceschini A., Wyder S., Forslund K., Heller D., Huerta-Cepas J., Simonovic M., Roth A., Santos A., Tsafou K.P. (2015). STRING v10: Protein–protein interaction networks, integrated over the tree of life. Nucleic Acids Res..

[76] Su G., Morris J.H., Demchak B., Bader G.D. (2014). Biological network exploration with Cytoscape 3. Curr. Protoc. Bioinform..

