# Supplementary material for: Genome-Wide Identification and Expression Profiling of Phosphatidylethanolamine-Binding Protein (PEBP) Genes in Helianthus annuus L
Source: Int J Mol Sci. 2025 May 11;26(10):4602. doi: 10.3390/ijms26104602 (PMC12110970; doi:10.3390/ijms26104602)
Supplement: Supplementary file 1 [file ijms-26-04602-s001.zip › Supplementary Materials.pdf]

Supplementary Legends

Supplementary Materials:

Table S1 Comprehensive characterization of 12 *PEBP* Genes in Sunflower;

Table S2 List of *AtPEBP*s, *SbPEBP*s and *OsPEBP*s Used in Phylogenetic Analysis with *HaPEBP*s, Including Their Classifications;

Table S3 Cis-Acting Elements Analysis in the 2 kb Promoter Regions of *HaPEBP* genes;

Table S4 Orthologous Gene Pairs within the *HaPEBP* Gene Family Identified in This Study;

Table S5 Tandem Duplication Block Types of 12 *HaPEBP* Genes;

Table S6 Homologous Relationships Between *HaPEBP*, *AtPEBP* and *OsPEBP* Genes;

Table S7 Subcellular Localization Prediction of *HaPEBP*s Based on MULocDeep;

Table S8 Three-Dimensional (3D) Structure Analysis of 12 HaPEBP Proteins;

Table S9 Ka/Ks Value Calculation Among the 12 *HaPEBP* Genes;

Table S10 Sequences of primers for subcellular localization and qRT-PCR.

Figure S1 Structural comparison of *HaPEBP* genes. Black lines indicate introns, while green blocks represent exons. Orange blocks denote UTR regions, with the 5' UTR highlighted in yellow and the 3'UTR in blue. The black scale indicates the sequence positions of the *HaPEBP* gene;

Figure S2 Multiple alignments of the conserved domains of HaPEBP proteins. The protein sequences are aligned using MAFFT. The alignment results are visualized in Overleaf. Conserved domains are highlighted with red frames.
