# Supplementary material for: Genome-Wide Identification and Expression Profiling of Phosphatidylethanolamine-Binding Protein (PEBP) Genes in Helianthus annuus L
Source: Int J Mol Sci. 2025 May 11;26(10):4602. doi: 10.3390/ijms26104602 (PMC12110970; doi:10.3390/ijms26104602)
Supplement: Supplementary file 1 [file ijms-26-04602-s001.zip › Table S10.pdf]

**Subcellular localization**	
**Gene ID**	Primers(5’ -- 3’)
**HaPEBP3**	Forward: ATGGCGAACGCAAGCGATGAGTTC<br>Reverse: TCTCCGTCGTCCACCAGATCCAC
**HaPEBP9**	Forward: ATGTCGAGGAGGGAGAGGGACCCGT<br>Reverse: ATTTACGGCCATCAGAACAGCCTC
**qRT-PCR**	
**HaPEBP3**	Forward: CATAATGACTTTAAGGTGCCTGGAT<br>Reverse: TACGGCCATCAGAACAGCCTCCC
**HaPEBP4**	Forward: TAACGATAGGGAAGTTAGCAACG<br>Reverse: GTTAGGGTCACTTGGACTTGGAG
**HaPEBP6**	Forward: CTCATGCCTAATCTCATTATCACC<br>Reverse: TGTCTCATAGCTTACAATTTCCCT
**HaPEBP9**	Forward: TGTTCCGGCAGTTGGGTCGACAA<br>Reverse: GTCGTCCACCAGATCCACTTTCAC
**HaTub**	Forward: CCGTCTTCACTTCTTTATGGTCG<br>Reverse: CAACTTCCTTGGTGCTCATCTTT
