# Supplementary figures and images for: Genome-Wide Identification and Expression Profiling of Phosphatidylethanolamine-Binding Protein (PEBP) Genes in Helianthus annuus L
Source: Int J Mol Sci. 2025 May 11;26(10):4602. doi: 10.3390/ijms26104602 (PMC12110970; doi:10.3390/ijms26104602)

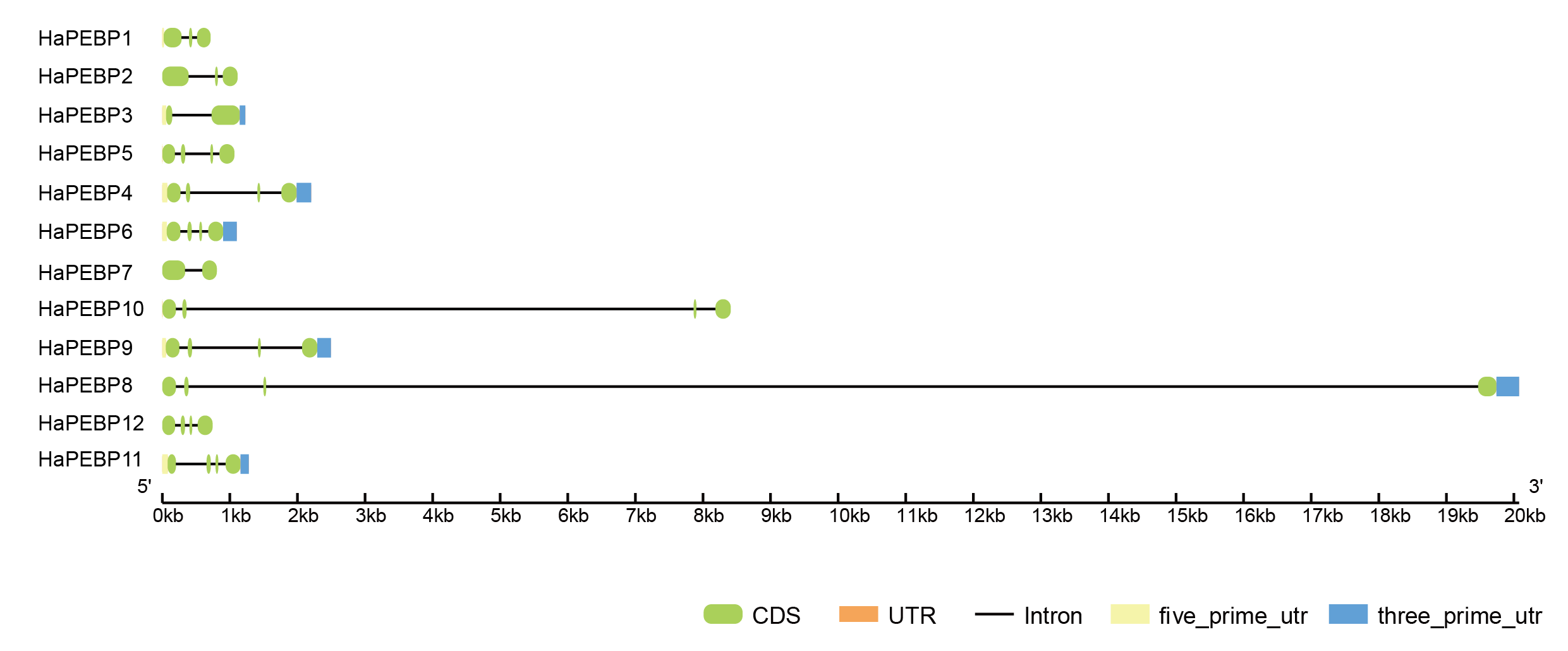

Supplement: Supplementary file 1 [file ijms-26-04602-s001.zip › Figure S1.png]

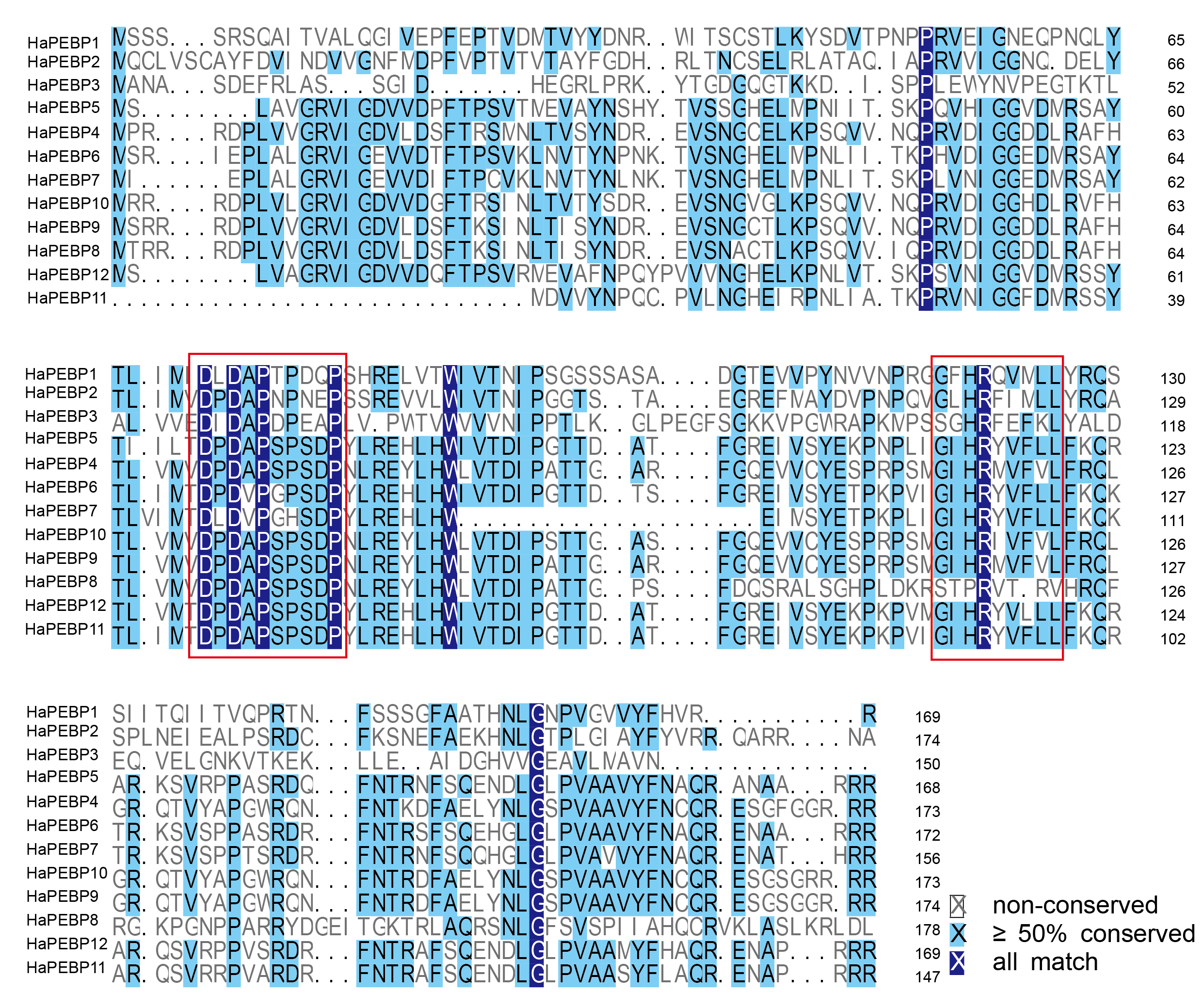

Supplement: Supplementary file 1 [file ijms-26-04602-s001.zip › Figure S2.png]
